# Nested Nanowell Arrays for High-Throughput Quantitative Analysis of Cytokines from Single Macrophages

**DOI:** 10.1021/acsnanomed.5c00149

**Published:** 2026-03-03

**Authors:** Claudius L. Dietsche, Lucien R. Stöcklin, Robert Strutt, Petra S. Dittrich

**Affiliations:** Department of Biosystems and Engineering, 27219 ETH Zurich, Schanzenstrasse 44, CH-4056 Basel, Switzerland

**Keywords:** microarray, single-cell analysis, protein secretion, high throughput analysis, cytokines, immunoassay, multiplexing

## Abstract

Macrophages play a critical role in the development of the tumor microenvironment (TME). Recruited macrophages in the TME differentiate into various phenotypes, each with a distinct profile of secreted cytokines. To describe and understand this large heterogeneity, we developed nested nanowell arrays for the multiplexed analysis of secreted proteins at the single cell level. The array consists of more than 100,000 wells on a cyclic olefin copolymer (COC) substrate. Each well contains seven smaller indents for cocapturing functionalized beads and can be operated with standard laboratory equipment such as pipettes and microscopes. The barcoded beads capture cytokines of interest and allow their quantification via sandwich immunoassays. We developed an image analysis tool and quantified 10 proteins secreted from single macrophages and investigated the effects of stimulation and drug treatment. We found that interleukin (IL)-1β, IL-8, and macrophage inflammatory protein 1α (MIP-1α) were highly secreted by more than 43% of the macrophages, with an increase of MIP-1α secretion under treatment with the chemotherapeutic drugs paclitaxel or docetaxel. Pairwise protein analysis confirmed cosecretion of IL-1β and IL-6 in macrophages stimulated with IL-4/IL-13, which were identified as part of a critical pathway in multiple myeloma before. We also demonstrate further multiplexing with three and four cosecreted proteins, together with assessing the cell viability as an additional parameter important for drug testing. In summary, we have shown that our nested nanowell arrays are an easy-to-use analytical tool for basic research, and we believe that it can be employed for diagnostics and personalized medicine, e.g., for investigation of cancer and immune cells from a biopsy or circulating tumor cells obtained from a liquid biopsy.

## Graphical abstract

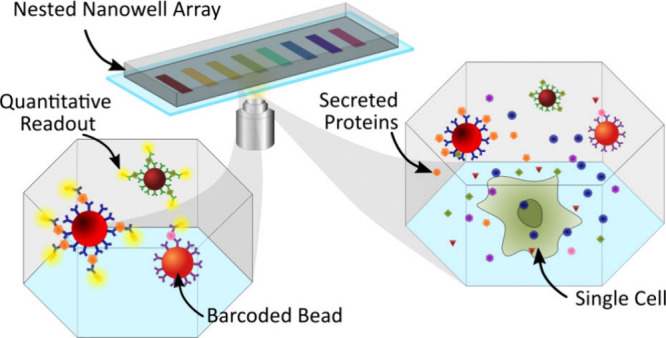

## 1 Introduction

Macrophages, use paracrine signaling of cytokines to activate various functions of the immune system.^1^ Specifically, in the tumor microenvironment (TME) macrophages play a crucial role.^2^ The highly plastic macrophages can respond to stimuli from their environment and adapt their phenotype accordingly.^1,3^ The heterogeneity of the TME results in diverse stimuli for individual macrophages, leading to the development of various phenotypes. Two extreme phenotypes of macrophages are well studied in the TME: the pro-inflammatory phenotype, also known as M1 macrophages, exert an antitumoral impact on tumor progression, while the anti-inflammatory phenotype, also known as M2 macrophages, promote tumor growth.^3,4^ Undifferentiated macrophages can be polarized *in vitro* to the M1 phenotype by stimuli such as lipopolysaccharide (LPS) and interferon y (INF-γ) whereas interleukin 4 (IL-4) and IL-13 stimulates macrophages toward a more M2-like phenotype.^3,5^ M1 macrophages secrete mainly pro-inflammatory proteins such as IL-1β, IL-6, IL-8, or tumor necrosis factor alpha (TNF-α) whereas M2 macrophage secrete cytokines such as macrophage inflammatory protein-4 (MIP-4/CCL18) or vascular endothelial growth factor A (VEGF-A).^3,5–7^ Macrophages are known to adapt to stimuli from the TME but also change their phenotype in response to various anticancer drugs.^4,8,9^ Recently, it has been shown that taxanes, a chemotherapeutic drug against various cancer types, change macrophages toward the pro-inflammatory phenotype, aiding tumor regression.^9^ As macrophages are heterogeneous, with numerous subtypes between the extremes, alterations in individual cells may be overlooked in bulk measurements. We therefore investigate the secretion profile of highly plastic macrophages at the single-cell level in response to anticancer drugs, which is crucial for developing innovative and efficient cancer treatments.

Microfluidic technology provides well-suited tools for single-cell analysis.^10,11^ Encapsulation of cells in small compartments, such as droplets^12–18^ or microchambers,^19–25^ has enabled the analysis of secreted proteins from individual cells. These compartments have volumes of nanoliters or even picoliters in which the proteins accumulate and can be quantified by immunoassays or other specific enzymatic assays. Approaches that are based on droplets, including hydrogel particles^26,27^ are designed for high throughput analysis, as the droplets can be generated continuously and at high frequency. Throughputs on the order of 10^4^–10^5^ cells per experiment enable robust statistical analysis. In contrast, microfluidic devices that incorporate addressable compartments (“microchambers”) by use of pneumatic valves typically reach lower throughput (∼10^3^ cells) but are generally offer higher multiplexing capabilities.^19–23,28^ The use of valves makes it possible to supply fresh medium or assay compounds or realize washing steps. On such devices, quantitative analysis of several proteins was realized by means of immunoassays. The parallel detection of several proteins, i.e., multiplexing, was achieved either by use of barcoded beads functionalized with target-specific antibodies or by spatial-controlled antibody immobilization.^21,23,28^ The use of polydimethylsiloxane as a microdevice material can also introduce artifacts into the results because many molecules are absorbed into the material or adsorbed on the surface. Another concept is based on open wells arrays^29–33^ which has high throughput and simplicity, as the cell suspension is pipetted directly on the structured surface. However, unlike other methods, complete isolation of the individual cells is not achieved, and preventing diffusion between wells becomes increasingly challenging as well density increases.

Here, we introduce several effective innovations that expand the versatility and performance of nanowell platforms while keeping the operation simple. We integrate a well array on a cyclic olefin copolymer (COC) plate with an ultrahigh density of 415 wells per mm^2^, in total 100,992 hexagonal wells. Smaller wells are incorporated within larger ones to facilitate the retention of magnetic, barcoded beads, even during washing procedures. Additionally, these ‘nested nanowells’ keep the beads separated at designated sites, and in the same focal plane, which support rapid imaging and streamlined data processing. This enables the identification of up to 10 different barcoded beads each functionalized with antibodies capturing cytokines of interest. The target cytokines can be selected from a large commercially available library (>600 analytes) and flexibly adapted prior to each experiment to address the specific scientific question being investigated. The wells are fully isolated during cell incubation, so that the proteins accumulate in the compartment. As in previous platforms, the capture of cells and beads are stochastic, but the large number of wells strongly increases the probability of capturing various combinations of single cells and single beads or bead pairs on the platform with high statistical confidence. We show that, in a single experiment, we can assess the secretion of the 10 different cytokines from polarized and unpolarized macrophages as well as drug-treated macrophages. Moreover, we can assess all possible 45 combinations of two proteins cosecreted from the same single cells. We also demonstrate further multiplexing with three and four cosecreted proteins.

## 2 Materials and Methods

### Wafer and Microarray Fabrication

The microarray is a patterned cyclic olefin copolymer (COC) substrate manufactured by hot embossing. A mold was required that was fabricated by standard lithography. The master mold was produced by use of two layers of SU8 (KAYAKU), each processed with a different mask design. The masks for the different layers were designed in AutoCAD (Autodesk) and were fabricated and purchased from Selba S.A. The first layer contained the pattern for the hexagonal cell traps (height: ∼45 μm), and the second layer contained the drainage system and magnetic traps with a diameter of 8.5 μm (height: ∼9 μm). The slight variation in the magnetic trap size results from light scattering during exposure of the SU8. Noteworthy, this difference does not affect the retention of the magnetic beads as can be seen in Figure 3E. For the first layer, SU8 3050 was spin coated (2500 rpm) on a dehydrated silicon wafer (MicroChemicals). After 10 min of pre-exposure baking at 95 °C, the SU8 layer was exposed with 175 mJ cm^–2^ in a mask aligner followed by a postexposure bake of 3 min at 95 °C. The second layer (SU8 3005) was directly spin coated on top of the first layer with a spin coating speed of 5000 rpm. The pre-exposure bake was reduced to 5 min, followed by exposure of the second layer with 450 mJ cm^–2^. The post exposure bake was again conducted for 3 min at 95 °C. Finally, the mold was developed in mr-DEV600 (Micro Resist Technology GmbH) for 15 min and post baked for 2 h at 160 °C. The final master mold wafer (Figure S4) was silanized by perfluorooctyl trichlorosilane (PFOTS, Merck).

The thermoplastic microarray was fabricated by a hot embossing method developed by Kling et al.^44^ The COC2008 foil (microfluidic ChipShop) with a thickness of 240 μm was placed above the SU8 master mold in a compact nanoimprinting (CNI) tool (NIL Technology) (Figure S5). To prevent breaking of the master mold, a silanized silicon wafer and a 1 mm thick aluminum plate was placed on top of the COC to homogeneously distribute the applied pressure by the CNI tool on the master mold. Two PTFE sheets prevented adhesion of the master mold and the aluminum plate to the CNI tool. The hot embossing was conducted with 6 bar at 145 °C for 5 min. The final pieces were cut out and sealed with scotch tape to reduce contamination.

Directly before the experiment was conducted, the eight areas (eight experimental conditions) were coated with antiadherence rinsing solution (STEMCELL) to reduce protein adhesion to the COC. This was achieved by pipetting 25 μL of the solution on each condition, placing the microarray in vacuum for 1 min to fill all wells, and incubating for 30 min.

### Magnet Holder Fabrication

The magnetic field was simulated in COMSOL Multiphysics (Comsol, Inc., Version 5.4) to achieve a homogeneous force on the magnetic beads in the nanowells without obstructing the field of view during imaging. The viewing holes in the magnet mount were designed to be larger than the dimensions of the individual condition to facilitate easy placement during the experimental procedure. After the optimal placement of the magnets was simulated, the holder was designed in SOLIDWORKS 2018 (Dassault Systèmes, Figure S6), and 3D printed with black poly carbonate (Ultimaker 3, UltiMaker). With the help of a plier, 45 quadratic neodymium magnets (3 × 4 × 8 mm, Webcraft AG) were inserted in the mount.

### Cell Culture

The THP-1 cell line (ATCC) was cultured in Roswell Park Memorial Institute (RPMI) media supplemented with 10% fetal bovine serum (FBS), 1xGLutaMAX and 1000 U mL^–1^ penicillin, and streptomycin (all from Thermo Fisher Scientific). To initiate the macrophage differentiation, THP-1 cells were cultivated for 24 h in 100 ng mL^–1^ of phorbol 12-myristate 13-acerate (PMA, Thermo Fisher Scientific; Table S1). After 24h, the cells were stimulated with either LPS (1 μg mL^–1^, Thermo Fisher Scientific) and IFN-γ (50 ng mL^–1^, Merck), IL-4 (50 ng mL^–1^, PeproTech) and IL-13 (50 ng mL^–1^, Thermo Fisher Scientific), paclitaxel (Taxol, 2ug mL^–1^, Merck), or docetaxel (Taxotere, 2ug mL^–1^, Merck) for 1 h (Figure S7).

The cells were detached by washing them in phosphate-buffered saline (PBS) before they were incubated for 15 min in TrypLE (Thermo Fisher Scientific). After successful detachment, they were stained with CellTrace Calein AM (Thermo Fisher Scientific) for 15 min. Before introducing the cells on the microarray, 3 washing steps in PBS were conducted.

### Experimental Procedure

For the immunoassays, we used a ProcartaPlex kit (Thermo Fisher Scientific, Table S2) and selected 10 different barcoded beads, functionalized with antibodies that specifically bind to the 10 tested proteins (VEGF-A, MIP-4, IL-1β, IL-6, IL-8, IP-10, TNF-α, OPN, MCP-1, MIP-1α). All different magnetic beads were mixed and diluted to achieve a final concentration between 10X to 25X (50X stock concentration). The beads were washed two times before use with the washing buffer provided with the kit. Twenty μL of the bead suspension was pipetted on each of the eight areas separately and carefully distributed by up and down pipetting of 10 μL multiple times. The assay washing buffer (1X) was used to wash the beads and remove all nontrapped magnetic beads from the microarray. The microarray was washed three times with the cell culture media (DMEM, Gibco) to remove all residues of the washing buffer. Afterward, 20 μL of the cells at an ideal concentration of 1.5 million cells per milliliter were pipetted onto the microarray. Depending on the cell confluency, the ideal concentration of cells was not reached, and the experiment was conducted with a reduced concentration. After 2 min settling time on a shaker, the microarray was immersed in HFE-7500 Engineered Fluid (FluoroChem). The residual media on the microarray was removed. The microarray was turned around and placed on a fully automated microscope (Nikon Ti2 eclipse). During the 2h incubation time, the cells were in an environmental chamber which provided an atmosphere of 5% CO_2_, a humidity of at least 80% and a temperature of 37 °C. All wells were imaged to quantify the CellTrace Calcein stain and therewith the viability of the cells during each experiment. After the microarray was taken out of the HFE-7500 container, all liquid was immediately removed by the use of filter paper (Whatman, Sigma-Aldrich) to prevent cross-contamination between wells. The microarray was put in 500 mL of DI water and placed in vacuum for 1 min to refill all wells. After rigorous washing with washing buffer (1X), 20 μL of biotinylated detection antibodies at a concentration of 1X were pipetted on the conditions and incubated for 30 min on a shaker at 150 rpm. This was repeated for the streptavidin-PE conjugate to stain the detection antibody. Finally, the reading buffer (1X) of the assay was pipetted on the microarray and a microscopy slide placed on the platform for the imaging of the barcoded beads.

### Bead Assay Characterization with Test Proteins

The protein dilution series for the calibration curves was prepared according to the ProcartaPlex protocol with the provided proteins in DMEM media at 10X. The beads were loaded on the microarray according to the experimental procedure protocol. The beads were washed three times with DMEM media before all liquid was removed with filter paper. On each condition, 20 μL of the protein dilution series was pipetted. The microarray was placed in a desiccator and a vacuum was applied for 1 min to remove all bubbles and guarantee a homogeneous filling of the nanowells. After 2 h of incubation at 37 °C and 5% CO_2_ the assay and readout of the beads was conducted according to the experimental procedure protocol. For the standard calibration curves, a 5PL fit was used to calculate the protein concentration as a function of the fluorescence intensity. The limit of detection (LOD) was established by adding three times the standard deviation to the mean of the control. The box and whiskers were obtained with standard algorithm in Matlab (whiskers: 1.5 × interquartile range (IQR)). The outliers are not shown.

### Cellular Metabolism and Survival Experiment

The cellular metabolism and survival experiment was conducted with THP-1 cells. The cells were stained with Calcein AM and the media contained 10% alamarBlue (Thermo Fisher Scientific) to show the metabolic activity of the cells. Dead cells were identified by the Calcein AM leaking from the cytosol into the chamber decreasing the ratio between the intracellular Calcein AM concentration and the well.

### Optical Setup

All images were taken with a fully automated Eclipse Ti2 (Nikon) microscope with an SOLA II (Lumencore) as a fluorescence excitation light engine. A DS-Qi2 camera (Nikon) and a 20x objective (Plan Apo VC 20X, NA:0.75, Nikon) were used for the acquisition of the images. Filter sets for DAPI, GFP, and mCherry were used for fluorescence imaging. For the readout of the beads, three custom filter sets were used: one for the assay readout of Phycoerythrin (PE: EX 530/40, DC 565, EM 611/75), and two for the barcode in the far-red spectrum (barcode 1 (B1): EX 628/32, DC 649, EM 670/30; barcode 2 (B2): EX 640/30, DC 649, EM 711/25). The light intensity and exposure time was fixed over all experiments and was set to 2%, 20%, 50%, 50%, 50%, 50% and 100 ms, 50 ms, 500 ms, 500 ms, 100 ms, 200 ms for DAPI, GFP, mCherry, PE, B1, B2, respectively.

### Data Evaluation

The images were evaluated with a custom MATLAB script with a graphical user interface. Briefly, the locations of the nanowells were determined for each image. In every well, the cells and the barcoded beads were detected, and the mean fluorescence intensities over the diameter of the beads in three channels (PE, B1, B2) were evaluated. The mean fluorescence intensity of each bead for phycoerythrin was used for further data analysis. When beads with the same barcode were entrapped in one well, the fluorescence intensity was added up. The fluorescence intensity of each bead was converted to the protein concentration based on the standard calibration curve evaluated for each protein. Beads below the background fluorescent intensity were assigned a concentration of 0 nM.

For Figure 5B and C, the average concentration was calculated by averaging the mean secreted protein concentration for every condition over all technical replicates. All beads above and below the LOD were considered. For the multiplexed data set in Figure 6 C and D, only beads with protein concentrations above LOD were considered.

The t-distribution stochastic neighbor embedding (t-SNE) algorithm was performed in MATLAB with the mean fluorescence data of the beads. The perplexity was set to 20 for Figure 7A–D and 10 for Figure 7E.

### Convolutional Neural Network

The convolutional neural network was created with the Deep Learning Toolbox from MATLAB. The network consists of three convolutional layers (3 × 3 kernel) followed by a batch normalization and ReLU layer. The number of filters used per layer was 8, 12, and 32. The stride length was fixed to 1 pixel. Between the convolution layer, a maximal pooling with a factor of 2 was performed. The validation accuracy was 97.8% with a data set of more than 10,000 beads.

### Statistical Analysis

All statistical evaluations were performed with MATLAB. The significant difference between distributions was evaluated with the two-sided student *t* test. If no indicator above a plot is shown, the two data sets are not significantly different (ns). **** *p* < 0.0001, *** *p* < 0.001, ** *p* < 0.01, * *p* < 0.05.

## 3 Results and Discussion

### 3.1 Development and Characterization of the Nested Nanowell Array

The developed platform contains 100,992 individual wells to analyze secreted proteins from single cells with an on-bead immunoassay (Figure 1). The wells are spatially separated into eight distinct areas that are suited to conduct different experimental conditions in parallel (Figure 2A). Between these areas, a drainage system comprised of 250 μm wide trenches is present. Each condition contains 12,624 hexagonal shaped wells with a side length of 20 μm. The wells are separated by a thin 20 μm thick wall on each side, resulting in a well density of 415 wells per mm^2^. Each large well contains seven small wells (ø = 7.5 μm) to capture and retain 6.5 μm in diameter magnetic beads. The size of the hexagonal-shaped wells is minimized to favor the capture of single cells while still accommodating at least seven indents for the small bead traps. These small traps retain the beads effectively and thereby allow thorough washing between assay steps. They also promote efficient bead separation, significantly reducing overlap and clustering during the automatic readout. This results in more precise and reliable readouts and thereby improves protein quantification. Additionally, the indent depth exceeds the bead diameter, which reduces the contact area between the functionalized beads and the trapped cells. This differs from previous approaches, where the cells are directly incubated on a functionalized surface, which potentially impacts their phenotype.

**Figure 1:**
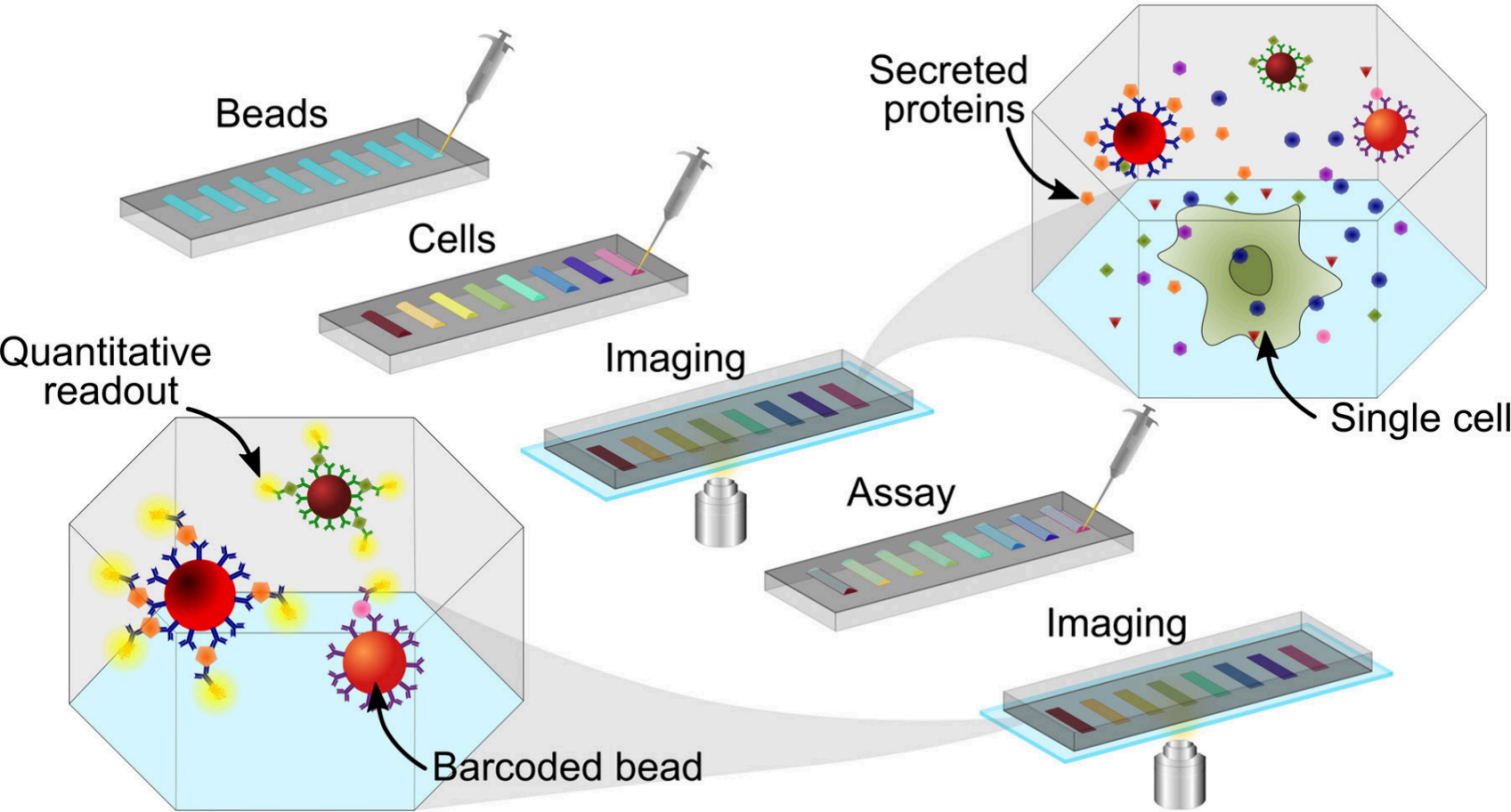
Conceptual abstract of the developed nested nanowell array device for the quantitative analysis of secreted proteins from single cells. Functionalized, barcoded magnetic beads are distributed in 100,992 wells. Individual cells are cocaptured with the functionalized beads and incubated in up to eight different conditions. The secreted proteins are captured on the beads and detected by means of a sandwich immunoassay, imaged by a fully automated microscope, and finally quantified by an image analysis software.

**Figure 2:**
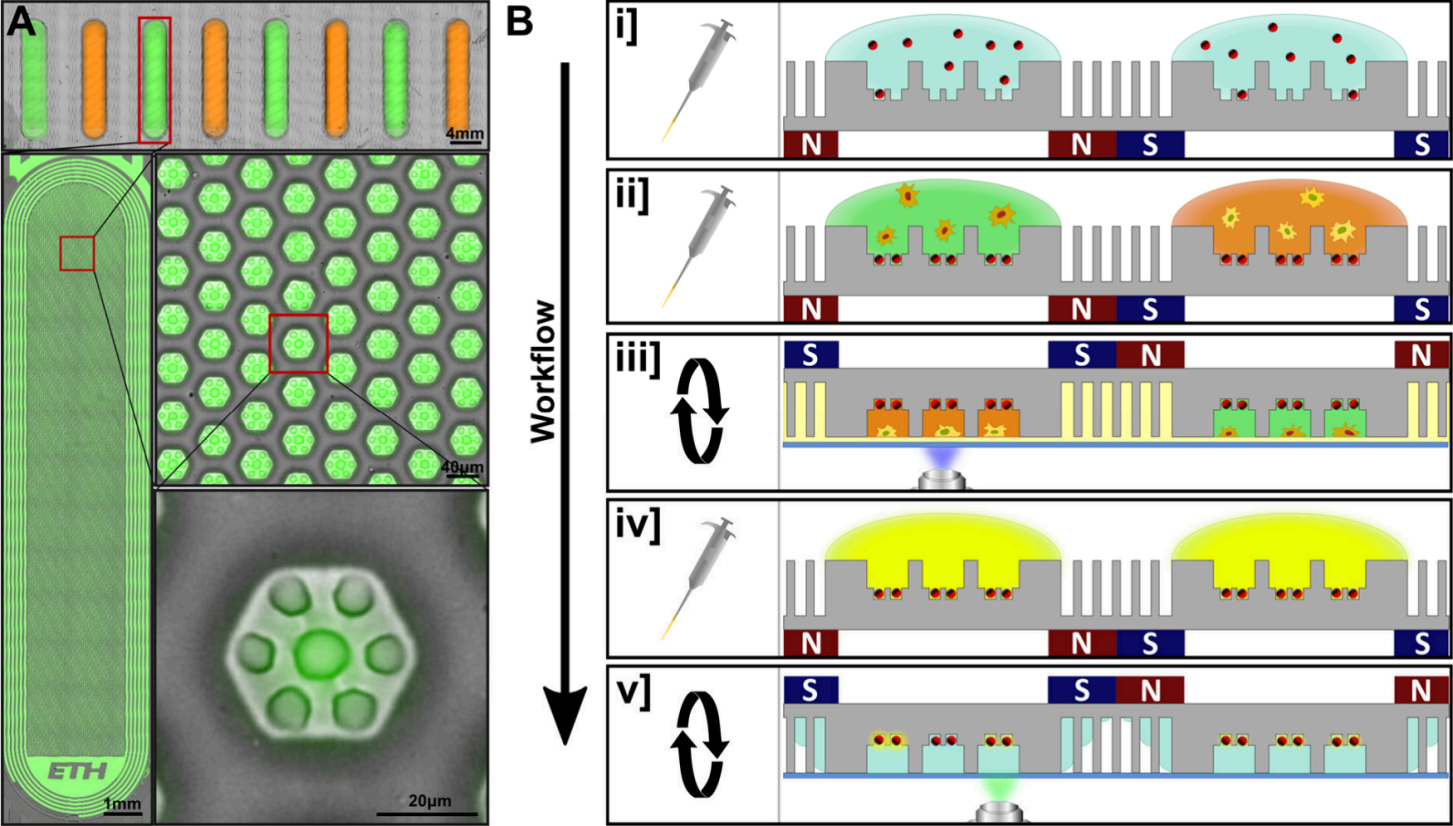
Design and workflow of the nested nanowell array device. (A) Microscopy images of the nanowell array. Each device contains in total 100,992 hexagonal wells which are designed to achieve a high well density and favor single-cell capture. Seven small indents in each well cocapture functionalized magnetic beads to immobilize and analyze secreted proteins. Up to eight different experimental conditions can be conducted at the same time. The scale bars represent 4 mm, 1 mm, 40 μm, and 20 μm. (B) The images of the cross-section of the wells depict the experimental procedure. (i) Commercially available magnetic beads are spread over the device and retained by a magnet placed beneath the microarray. (ii) The cells are pipetted on each condition area and captured by the hexagonal cell traps. (iii) The wells are “sealed” by placing the nanowell array in an HFE oil bath and removing the excess aqueous solution. The device is turned around and imaged on an inverted microscope. (iv) The second phase is removed, and the device is turned back to perform the immunoassay with the magnetic beads. (v) Finally, the beads are imaged in a fully automated procedure.

Initially, the beads are loaded onto the entire microarray by pipetting (Figure 2B). A magnet, which is placed under the array, prevents the magnetic beads from escaping the magnetic traps. After washing, cells exposed to different treatments are pipetted onto different subsets of wells. The microarray is submerged in a fluorinated oil bath, and the excess aqueous phase is removed with a pipet. Due to the slightly hydrophobic properties of COC (∼94 ± 4°) and the surface tension of the aqueous liquid, excess liquid can be removed by pipetting through the oil layer. Once the excess liquid is removed, the interface of the second phase sealed the hexagonal wells. From this moment on, all cells are isolated inside of individual wells. For the incubation and imaging of the cells, the microarray is turned and placed on a microscope. To perform the immunoassay after the incubation, the second phase (oil) is removed, all cells are washed away, and the mix of detection antibodies and subsequently the readout fluorophore (phycoerythrin, binding via streptavidin–biotin linkers to the antibody) are pipetted on the microarray. Between each step, the beads are thoroughly washed three times. Finally, the beads are imaged again upside down.

We characterized the microarray regarding well occupancy with beads and cells; the bead retention during washing steps; cross contamination between conditions and individual wells; and the cell viability and metabolic activity on the platform (Figure 3). The number of captured beads per well increased with the bead concentration following the Poisson distribution (Figure 3A). At the maximum tested concentration of 5.5 × 10^6^ beads per milliliter, 90% of all wells contained at least one bead with the highest probability (20%) of having four beads in one chamber (Figure 3B). We observed the same dependence for cell capture (Figure 3C). At a concentration of 1 × 10^6^ cells per milliliter, a third of all wells contained a single cell. At the highest concentration of cells (4 × 10^6^), 93% of all wells contained at least one cell and up to 69% at a concentration of 1 × 10^6^ (Figure 3D). In the following experiments, we used bead concentrations of 1.25 × 10^6^ to 3.13 × 10^6^ beads per milliliter and ideally, a cell concentration of 1.5 × 10^6^ cells. The cell concentration was chosen to maximize single-cell occupancy of the wells based on Poisson statistics (λ = 1, corresponding to a probability of 37%). At this concentration, many wells contain more than one cell; however, using automated image analysis, the number of cells in each well was determined and wells containing more than one cell were excluded from further analysis. The final cell concentration may have varied slightly due to yield variations after THP-1 treatment, resulting in an overall capture efficiency of approximately 27% across all experiments. The wells typically contained between 1 to 5 beads of random type ensuring that 14% (∼14 000) of the wells contained a macrophage and at least one bead.

**Figure 3:**
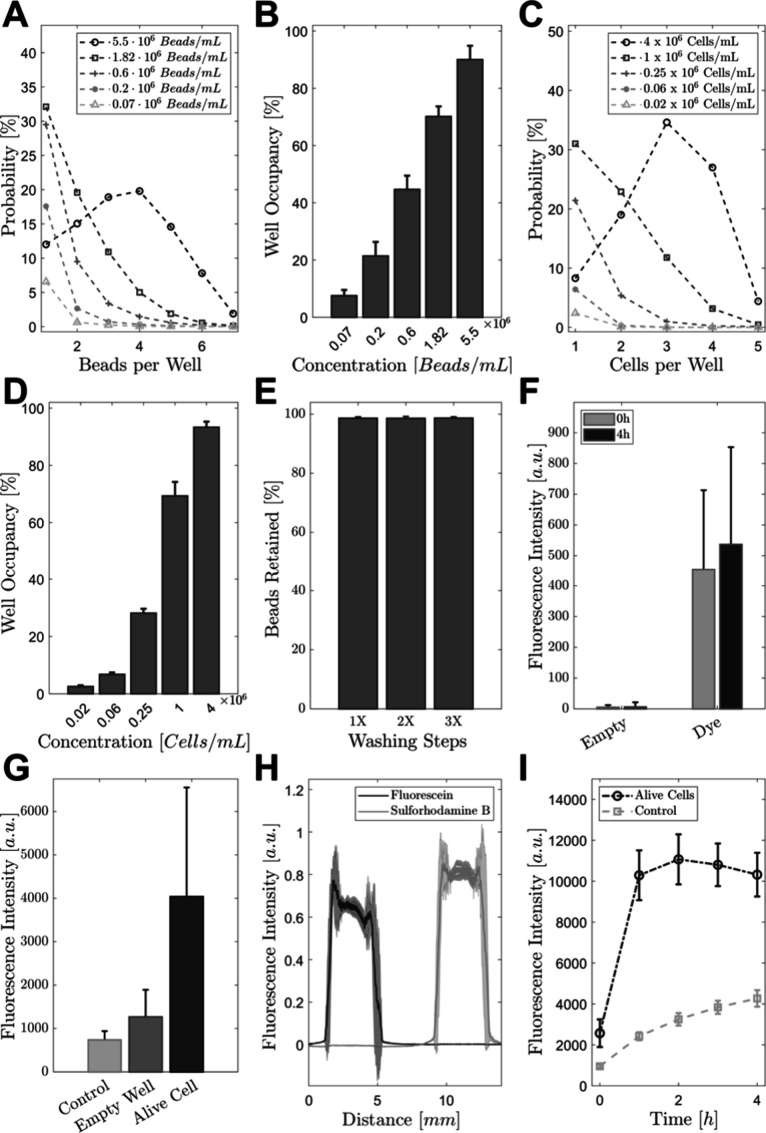
Platform characterization. (A, B) Probability of the captured bead number per well (A) and probability of occupied wells with at least one bead as a function of the bead concentration (B) (*n* ≥ 425). (C, D) Probability of the captured cell number in each well (C) and probability of occupied wells with at least one cell as a function of the cell concentration (D) (*n* ≥ 271). (E) Bead retention after several washing steps (*n* ≥ 1387). (F) Calcein AM intensity measured over 4 h to show well-to-well isolation. Empty wells contain no cell whereas wells with dye contain dead cells stained with Calcein AM (N_technical_ = 8, *n* ≥ 29,002). (G) Fluorescence signal of IL-8 was measured in pure media (control), wells containing no cells (empty), and wells containing metabolic active cells (alive cell) (*N* = 1, *n* ≥ 194). (H) Depicted is the normalized mean intensity of neighboring conditions filled with two different fluorophores (*N* = 1, *n* = 4). (I) Alamar Blue intensity shown for wells containing alive cells and no cell (control) for the duration of 4 h (*n* ≥ 2536). The error bars depict the standard deviation in all subplots. *N*_technical_ = 3 in all subplots if not noted differently.

Since washing steps were included in the protocol, we also confirmed that beads remained in the wells after each step (Figure 3E). The magnetic bead traps successfully retained around 99% of the magnetic beads during each washing step.

Another critical parameter of nanowell arrays is the isolation of each well, preventing cross-contamination between a well and its neighboring wells. We tested the cross-contamination between wells by staining the cytosol of cells with Calcein AM (Figure S1). Upon cell death, the cell membrane becomes permeable to Calcein AM, and the fluorescent molecules are released into the surrounding. If the well is sealed, then the fluorescence remains contained within the well. However, if the well is not sealed, fluorescent molecules diffuse into neighboring wells. We examined the fluorescence intensity of wells containing no cell (empty wells) and wells containing membrane permeated cells (dyed wells) over the duration of 4 h (Figure 3F). During this time, no leakage from dye containing wells to empty wells was observed.

Additionally, we analyzed wells containing highly secreted cells and neighboring empty wells (Figure S1). The neighboring empty wells had a protein readout below the limit of detection (LOD) which implies that the proteins are isolated in each well and show no cross contamination to adjacent wells. However, evaluating all empty wells of an experiment results in a background slightly higher than a control (pure media) condition (Figure 3G). Due to our sensitive system, the immunoassay detects proteins secreted by the cells during the short washing before they are isolated into the microarray wells. To compensate for this, we corrected the fluorescence intensity by subtracting the background signal in empty wells.

We also confirmed that the drainage channel system between the 8 subsections efficiently separated the 8 reaction conditions. Imprecise pipetting or handling errors can cause liquid to spill over into adjacent areas. To facilitate easier handling of the platform, we incorporated drainage channels that transport excess liquid away from neighboring conditions to the sides of the platform. To ensure that each condition is properly separated, we tested the platform by filling alternating conditions with two different fluorophores. (Figures 2A and 3H). The fluorophores were contained in the drainage system around the conditions but did not spread to adjacent conditions due to the design of the drainage system.

Finally, we investigated the metabolic activity of single cells within the wells. Our results showed that wells containing cells (i.e., those with intact membrane) exhibit an increased alamarBlue fluorescence intensity compared to empty wells (Figure 3I). This increase occurs because nonfluorescent resazurin is reduced to fluorescent resorufin by living cells. This shows that the cells captured in the wells are still metabolically active and secrete the proteins of interest.

### 3.2 Characterization of Bead-Based Sandwich Immunoassay

The commercial bead assays (ProcartaPlex, Thermo Fisher Scientific) were characterized on nested nanowell arrays. From over three hundred different targets to analyze with the beads assay, we chose 10 proteins, VEGF-A, MIP-4, IL-1β, IL-6, IL-8, IP-10, TNF-α, OPN, MCP-1, and MIP-1α (Figure 4), which are implicated in the polarization of macrophages. The fluorescence intensity combination of two far-red fluorophores embedded in the beads resulted in the barcode, which defined every bead type (Figure 4A, C). The barcode correlates to the primary antibody conjugated on the surface of the bead and the protein concentration was determined by the fluorescence intensity in the yellow spectrum to capture the phycoerythrin fluorescence. To streamline barcode classification, a convolutional neural network was trained (Figure 4B) on thousands of bright field and fluorescence images of beads to assign each bead to the corresponding protein (Figure 4C, D). We recorded calibration curves for the proteins and determined the LODs, which strongly depended on the analyzed protein, e.g., 0.055 nM for MIP-1α and 0.864 nM for TNF-α. In absolute number of molecules, the LOD is low with <1450 molecules for MIP-4, IL-1β, and MIP-1α (Figure S2).

**Figure 4:**
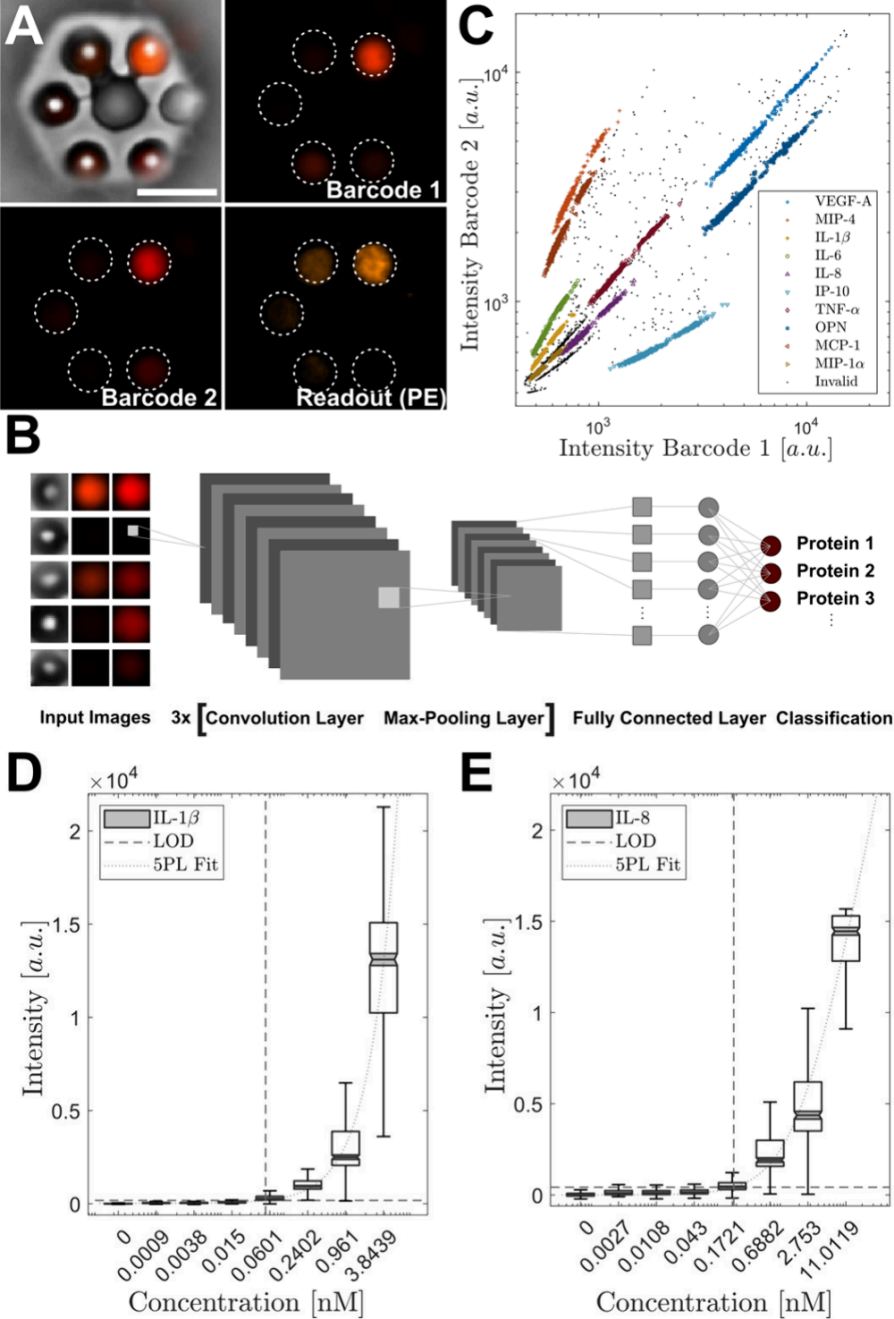
Characterization of the bead assay. (A) Images of a well containing five different bead types. The top left image shows the overlaid brightfield image and fluorescence images in three wavelength regions while the other fluorescence images show only one wavelength region. Two fluorophores in the red spectrum define the barcode of the beads. The fluorophore tagged to the detection antibody (phycoerythrin, PE) is detected in the yellow wavelength region and used to quantify the respective protein concentration in the well. Scale bar: 20 μm. (B) The barcode of each bead is determined by a convolutional neural network (CNN) which classifies each bead based on its brightfield and the two fluorescence images in the red wavelength region. (C) Classification of 10 different proteins from one experiment based on the trained CNN. (D, E) Example calibration curve of the proteins IL-1β and IL-8 (*N* = 1, *n* = 248). The LOD is calculated by adding three times the standard deviation to the mean of the control (at concentration 0 nM). *N* = number of experiments, *n* = number of analyzed beads.

### 3.3 Secretion Profile of Single Macrophages

Next, we analyzed proteins secreted by individual macrophages. We compared unpolarized MΦ macrophages with polarized macrophages, derived from MΦ and stimulated into the pro-inflammatory phenotype (M1, by LPS/INF-γ treatment) and the anti-inflammatory phenotype (M2 by IL-4/IL-13 treatment). Additionally, we treated MΦ with two drugs against tumors, paclitaxel (PTX, Taxol) and docetaxel (DTX, Taxotere).^8,9^ The cells were pipetted onto a distinct area of the bead-loaded microarray to capture single cells within the hexagonal wells. After 2 h incubation, the concentrations of the 10 target proteins were determined. We also assessed the viability for each cell using Calcein, which fluoresces in the green spectrum and does not interfere with the other fluorescence measurements. Wells that did not contain exactly one cell were excluded from the analysis and were not considered for the data evaluation.

Example raw data for the 10 proteins secreted from single unpolarized macrophages (MΦ) are displayed in Figure 5A. For certain proteins, such as IL-8 (59%), IL-1β (57%), and MIP-1α (59%), the measured intensity is above the LOD for most cells. Despite the relatively high LOD of the OPN and the low secretion of VEGF-A, MIP-4, IL-6, IP-10, TNF-α, and MCP-1, we could still obtain data points above the LOD for these cytokines. By using the calibration curves (Figure S2), the fluorescence signals were converted to concentrations. The overview is given in Figure 5B, and selected proteins with significant changes are displayed in Figure 5C. For these proteins, we additionally determined the percentage of cells that secreted the selected protein above the LOD (Figure 5D).

**Figure 5:**
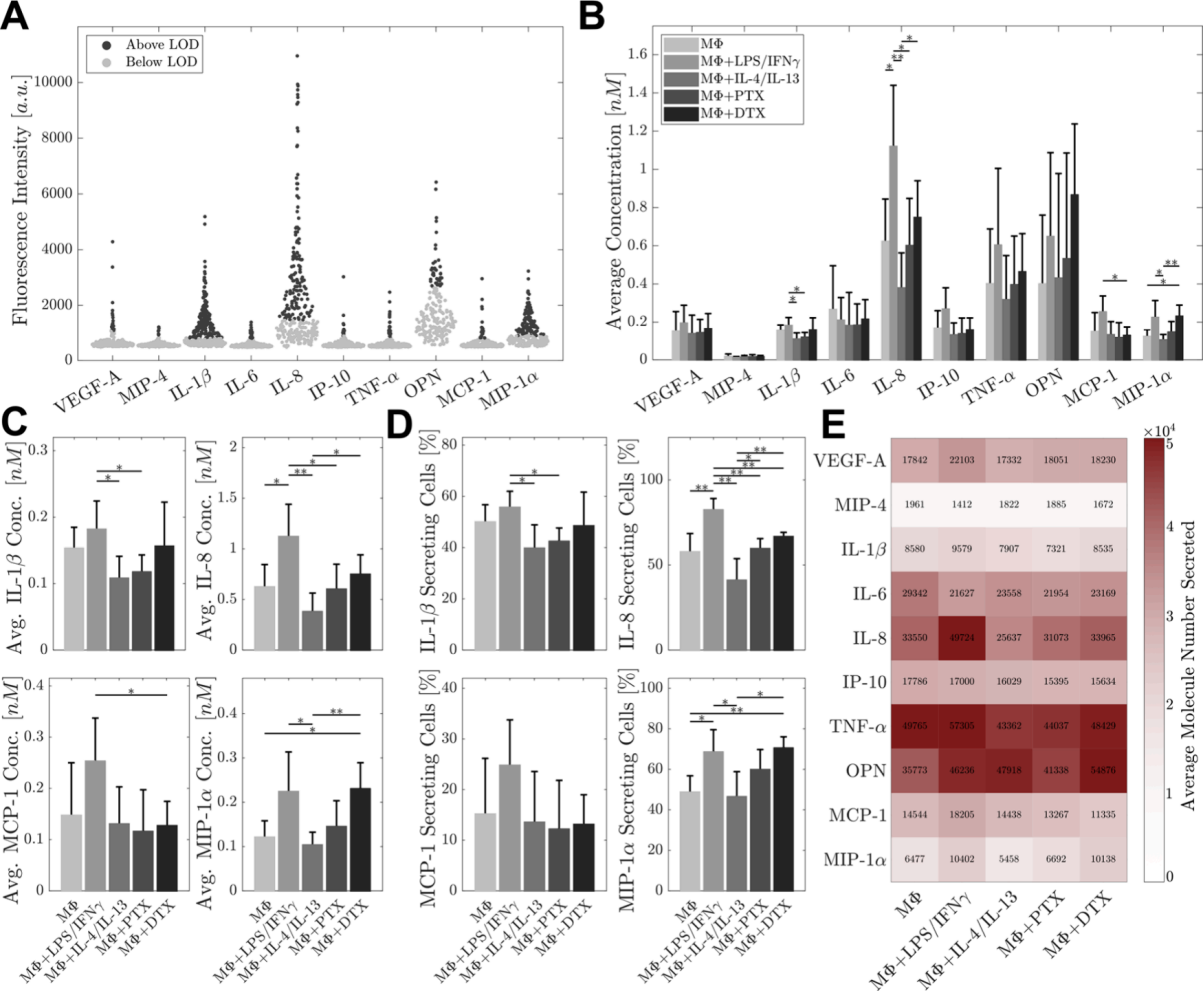
Protein secretion from single cells. (A) The readout fluorescence intensities measured for the 10 target proteins secreted by unpolarized macrophages (MΦ) from one experiment. Every data point represents the readout of one single cell (*N* = 1, *n*_total_ = 2520). (B) The average protein concentration secreted by single cells over 2 h was evaluated for all target proteins. Unpolarized macrophages and macrophages treated with different stimuli were investigated (*N* = 3, *n*_total_ = 41,753). (C) The average secreted protein concentration depicted for selected proteins. (D) The percentage of cell secreting over the LOD shown for selected proteins and different stimulation conditions. (E) The average number of secreted molecules for each protein and condition averaged over all single cells of all measurements (*n* ≥ 401 for every value). *N* = number of experiments, *n*_total_ = number of analyzed cells. The error bars depict the standard deviation of the sample.

The secretion of the pro-inflammatory cytokine IL-8 was significantly higher for M1 differentiated macrophages (1.15 nM) and significantly more cells were secreting IL-8 (84%) compared to MΦ macrophages (0.63 nM, 58%). Other pro-inflammatory proteins such as MCP-1, MIP-1α, and TNF-α were also secreted at higher levels, although statistical analysis indicated that the increase was not significant (α = 0.05). The higher secretion of pro-inflammatory proteins aligns with previous observations for macrophages treated with LPS and/or INF-γ.^23,34–36^ In contrast, M2 differentiated macrophages exhibited lower secretion of IL-1β, IL-8 and IP-10 compared to MΦ macrophages. The number of cells that secreted above the LOD reflected the same relative results as those of the concentration measurements. In other words, if a cytokine was highly secreted, this secretion pattern could be found in many cells (Figure 5D). Additionally, we calculated the average number of secreted molecules over two h for all proteins and stimulation conditions (Figure 5E). Among these proteins, IL-8, TNF-α, and OPN were consistently among the most highly secreted across all stimulation conditions, with the highest secretion of IL-8 and TNF-α observed in M1 macrophages (up to 57,000 molecules secreted within 2 h).

The MIP-1α is an inflammatory chemokine regulating the immune cell trafficking around the TME and is associated with a pro-tumoral function in the TME.^37–39^ MΦ macrophages treated with DTX exhibited a significant increase in the number of secreting cells and MIP-1α secretion as compared with the MΦ macrophages. However, we found no increase in IL-8 or IL-1β secretion, as observed in previous bulk experiments.^8^ A weak increase in MIP-1α secretion was also observed by MΦ macrophages treated with PTX (Figure 5C/D). A similar increase in MIP-1α secretion was measured by Wanderley et al. after two doses of PTX over 48 h.^9^ We speculate that the observed increase in MIP-1α secretion following paclitaxel and docetaxel treatment in the absence of detectable changes in IL-8 or IL-1β, may be related to the isolation of individual cells in the single-cell assay. Under these conditions, paracrine signaling between neighboring cells is largely absent, which can otherwise amplify or coordinate cytokine secretion in bulk populations. As a result, single-cell measurements primarily capture the direct response of individual cells to the applied stimulus without additional modulation by cytokines secreted from surrounding cells. This suggests that differential secretion of the analyzed cytokines in bulk populations may partly depend on secondary, population-level signaling that is reduced under single-cell conditions. Based on our findings, investigating a combinatorial drug treatment aimed at reducing the secreted concentration of MIP-1α during paclitaxel or docetaxel treatment would be of great interest.

### 3.4 Pairwise Analysis of Secreted Cytokines

The pairwise secretion analysis of single cells revealed correlations between different proteins. With 10 different bead types, 11’440 bead combinations in each well were possible (Figure S3), where each combination had only a small probability of occurring. Therefore, we focused on pair and triplicate bead combinations, which resulted in statistically meaningful data for each combination. With approximately 14% of wells containing two beads of different types coencapsulated, we could distinguish whether two proteins were cosecreted above the LODs (+/+),one protein was secreted (±, ∓), or if both proteins were not secreted (−/−) as shown in the scatter plot for the pair IL-1β/IL-8 (Figure 6A). The percentages of cells in each area are displayed in Figure 6B together with the protein pairs MCP-1/IL-8 and MIP-1α/IL-8. These graphs show that the polarization of macrophages into M1 or M2 led to large changes in the cosecretion pattern, while the drug treatment of macrophages had less effect on this pattern. Specifically, stimulation to M1 led to a high percentage of cells cosecreting MIP-1α/IL8 (76.9%), whereas only 31.6% of M2-stimulated cells cosecreted these proteins. MCP-1 is always cosecreted with IL-8 in M1 cells, but there was a large fraction of cells that secreted only IL-8.

**Figure 6:**
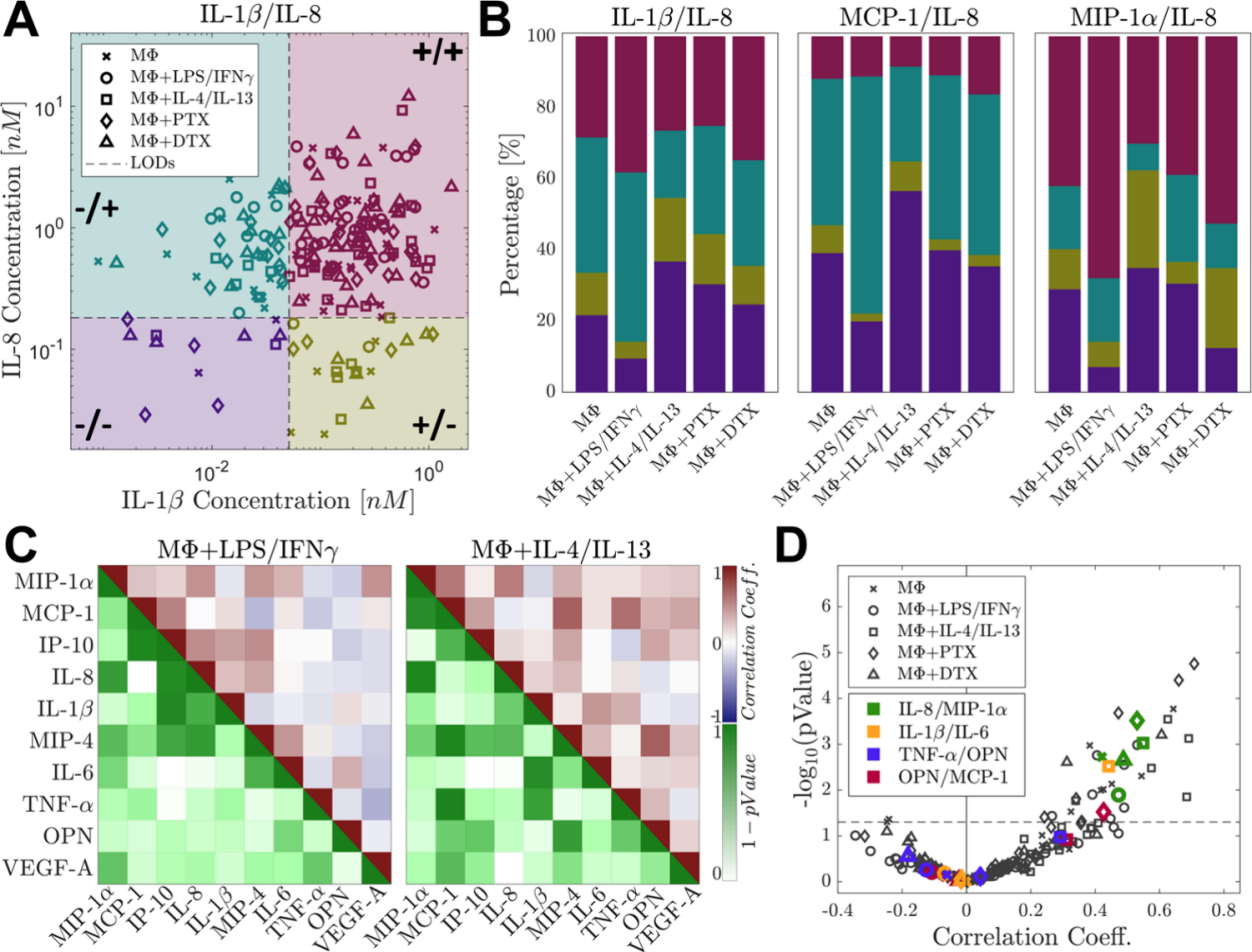
Pairwise correlation analysis. (A) IL-1b secretion plotted versus IL-8 concentration of single cells. Each cell is classified by its secretion pattern: secreting both proteins (top right), secreting only one (top left, bottom right), or none (bottom left) (*N* = 3, *n* = 376). (B) The percentage of cells in each classification region shown for different stimulations and selected protein pairs. (C) The pairwise correlation matrix and their significance for MΦ+LPS/INFy and MΦ+IL-4/IL-13 macrophages shown for all measured proteins. (D) Volcano plot of the correlation coefficient between the proteins. The significance level of 0.05 is shown by the dashed line.

The ratio of cells secreting only MIP-1α compared to those secreting only IL-8 varied significantly, shifting from approximately 1:1 for unpolarized macrophages and M1 phenotype to 14:1 for M2, reflecting the decrease of IL-8 secreting cells for the M2-stimulation as observed in Figure 5C. Conversely, for IL-1β/IL-8, M1 had a ratio of IL-1β to IL-8 secreting cells of 1:11. While this classification is dependent upon the LOD of our platform, it serves as a qualitative method to evaluate changes in percentage within each region based on macrophage stimulation.

The pairwise correlation coefficient matrix shows the secretion relationship among all proteins for each stimulation condition (Figure 6C). Remarkable were the changes in the correlations of TNF-α, VEGF-A, and OPN with most other proteins upon macrophage stimulation. These correlations shifted from mainly negative for M1 phenotypes to largely positive for M2 phenotypes, indicative of cosecretion of these proteins. The elevated levels of all these proteins in the TME are associated with promoting tumor progression.^40–42^ To visually represent the various correlations and their significance, we generated a volcano plot where significant protein pairs are clearly depicted (Figure 6D). The pro-inflammatory protein pair, IL-8/MIP-1α, was positively correlated independent of the stimulation condition. However, we found a significant positive correlation between IL-1β and IL-6 by M2 (0.55) compared to no correlation (0.018) with M1. Previous studies have demonstrated that IL-6 acts as a pro-tumorigenic agent and is induced by IL-1β.^43^ In a phase II study, it has been shown that inhibiting the IL-1β/IL-6 pathway improves overall patient survival in multiple myeloma patients with a combinatorial treatment of Anakinra (IL-1 receptor antagonist) and dexamethasone (anti-inflammatory).^43^ We observed similar changes in correlation in the pro-tumoral protein pairs TNF-α/OPN and OPN/MCP-1. Interestingly, treatment with paclitaxel led to an even higher correlation between OPN/MCP-1 compared to M2 macrophages, suggesting that the OPN/MCP-1 could be a potential drug target for combination therapy with taxanes.

### 3.5 Multiplexed Secretion Analysis

Our platform facilitates the pairwise analysis of cosecreting proteins from 10 different protein targets. Highlighting the flexibility of the device, which allows for the simple exchange of target proteins by adjusting the bead cocktail used during the assay, we decided to focus on proteins significantly secreted by macrophages (IL-1β, IL-8, TNF-α, and MIP-1α) to demonstrate an increased multiplexing capacity. To visualize the result, the secreted proteins were mapped onto a two-dimensional plane using the t-distribution stochastic neighbor embedding (t-SNE) algorithm (Figure 7). Notably, the pro-inflammatory M1-type macrophages (red) display distinctive clustering compared to macrophages under other stimulation conditions. This clustering phenomenon was consistent across all of the protein combinations. This distinct clustering is due to the higher secretion of the target proteins, particularly IL-1β, IL-8, by the M1 phenotype compared to the M0 and M2 phenotypes, which are more heterogeneous. Conversely, no clustering was observed among unpolarized (MΦ, blue), anti-inflammatory (M2, yellow), and docetaxel-treated (green) macrophages. This is primarily due to the lower secretion levels of the targeted proteins, making clustering of these phenotypes more challenging. Regardless of the reduction in the number of target proteins, the occurrence of wells with a single cell and all four beads remained relatively low (n = 57). Nevertheless, the visualization reaffirmed the clustering of M1-phenotype cells, while macrophages exposed to other treatments and unpolarized macrophages exhibited a scattered distribution (see Figure 7E).

**Figure 7:**
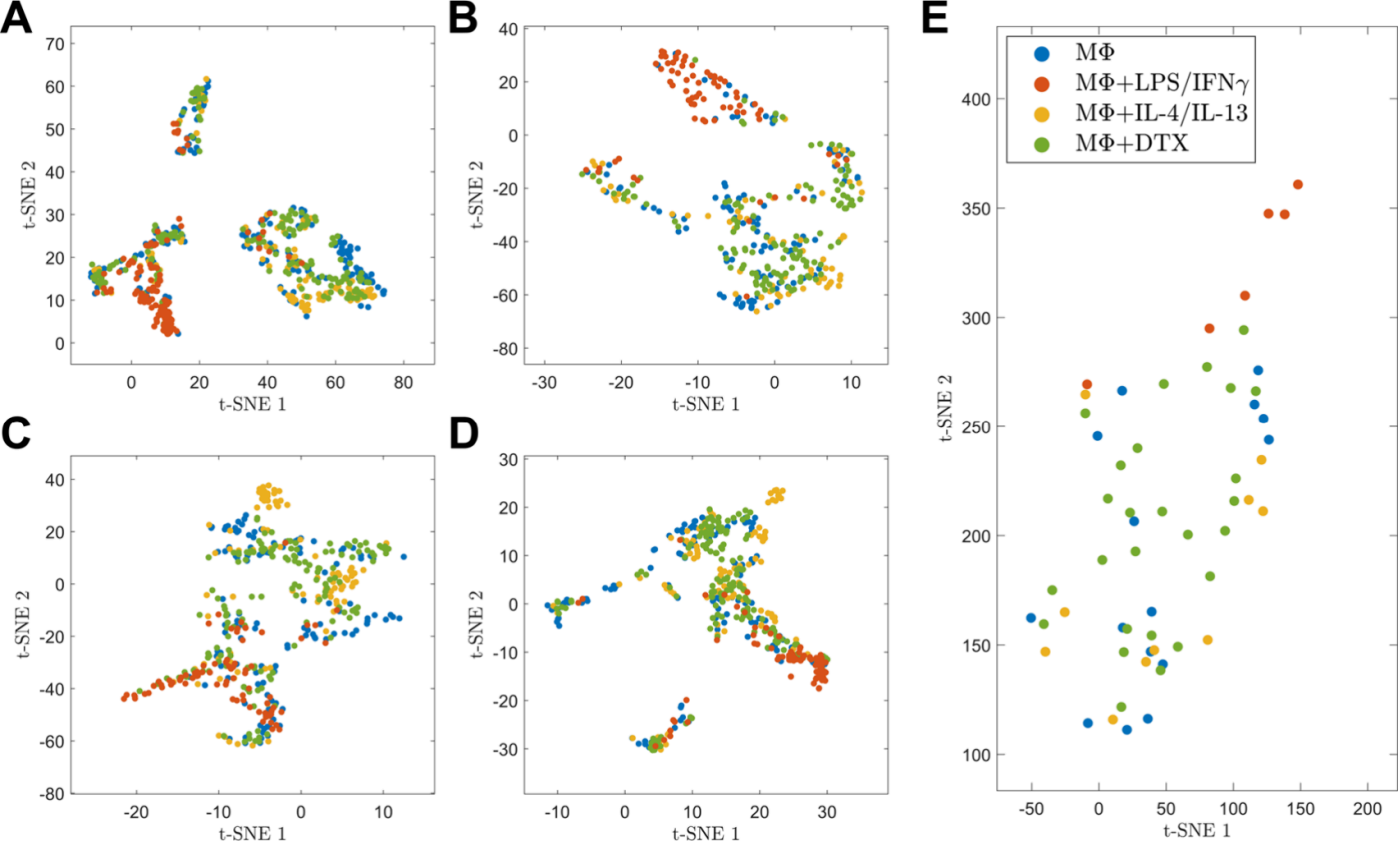
Multiplexed protein secretion from single cells. (A–D) The t-SNE algorithm was used to generate a two-dimensional representation based on the secretion of IL-1β, IL-8 and TNF-α (A, *n* = 555), IL-1β, IL-8, and MIP-1α (B, *n* = 360), IL-1β, TNF-α, and MIP-1a (C, *n* = 442), and IL-8, TNF-α, and MIP-1α (D, *n* = 445). Each data point originates from an individual cell and the color indicates the stimulation condition. (E) T-SNE representation of four proteins (IL-1β, IL-8, TNF-α, and MIP-1α) (*n* = 57). *N* = 2 for all subplots.

To increase the multiplexing capacity of the device, more nested nanowells, i.e., ‘bead traps’ per well are required, which could be achieved by using a more precise fabrication technique. For example, three traps per edge plus one central trap would lead to 19 bead traps. Such an approach would substantially increase the number of possible bead combinations, while maintaining the same overall footprint. At the same time, a moderate increase in the well size could further enhance the multiplexing capacity. For example, an increase in well size of approximately 50% would nearly double the number of nested nanowells to 37 per well. However, increasing the number of nested nanowells directly correlates with the number of beads needed per experiment and would require upconcentration of bead suspensions of stock solutions from the commercial providers. In addition, increasing the well size introduces trade-offs, including a larger device footprint and a higher probability of capturing more than one cell per well.

With the developed platform, we are able to quantify cytokines secreted by individual immune cells, which is relevant for translational medicine since secreted proteins impact disease outcomes such as cancer or autoimmune disorders. Multiple studies have shown that small subsets of cells, which are masked in bulk measurements, can strongly influence overall disease progression or serve as markers for therapy response.^45–47^ In particular, with the increasing use of targeted therapies and precision medicine approaches, technologies that can link protein secretion profiles to disease states are of growing interest. By enabling multiplexed and quantitative single-cell secretion analysis using standard laboratory equipment, the platform supports applications such as ex vivo drug testing and profiling of tumor associated macrophages.

## 4 Conclusion

We developed a highly parallelized, versatile, and user-friendly platform capable of analyzing a large population of single cells. We demonstrated that the nested nanowell arrays could detect low concentrations of proteins (down to 1200 molecules) in each well through an on-bead sandwich immunoassay. Moreover, the developed platform facilitated the study of macrophage polarization using 10 secreted proteins and enabled the profiling of secretion patterns for unpolarized and polarized macrophages as well as macrophages treated with anticancer drugs. We observed that the cytokines IL-1β, IL-8, and MIP-1α are secreted the most abundantly. Stimulation with LPS/INF-γ to an M1 phenotype resulted in a 76% increase in IL-8 secretion. Additionally, we demonstrated that the treatment of macrophages with taxanes (paclitaxel and docetaxel) resulted in an increase in MIP-1α secretion. Moreover, we studied the pairwise correlation between 45 protein pairs, identifying significant protein correlations. Specifically, our results indicate the importance of the cosecretion of IL-1b/IL-6 which is a target in current phase II study and of OPN/MCP-1 as a potential drug target for combinatorial treatment with taxanes. Multiplexed analysis of four proteins unveiled a distinctive secretion pattern in pro-inflammatory macrophages when compared to other investigated phenotypes, particularly concerning the proteins IL-1β, TNF-α, and MIP-1α. Further multiplexing requires increasing the number of magnetic bead wells within each well. This can be achieved by increasing the hexagonal well size, resulting in a higher ratio of functionalized beads to single cells and enhancing the platform’s multiplexing capability. Although the higher multiplexing capability is limited, the platform is well-suited for measuring the secretion of over 15,000 cells per experiment with easy and user-friendly handling. The beads are commercially available, so that target proteins can be easily changed to obtain different combinations. No special preparation of the platform, e.g., immobilization steps for the capture antibodies, is required. In summary, our results underline the versatility of our method for obtaining large single-cell data sets and improve our understanding of immune cell signaling with and without drug exposure in the context of cancer biology. Other drugs of interest could be immune-modulating agents, checkpoint-related modulators, or small-molecule inhibitors targeting inflammatory signaling pathways. We believe that it has the potential to support translational research and guide personalized treatment strategies by linking cell secretion to clinical outcomes.

## Supplementary Material



## Data Availability

The data that support the findings of this study are available from the corresponding author upon reasonable request.

## Notes

Additional characterization data, complete set of calibration curves, combination equations, height profile of the microarray, details of the fabrication procedure, crosstalk experiment, and complete list of proteins and ProcartaPlex assays (PDF)
