# Supplementary material for: Nested Nanowell Arrays for High-Throughput Quantitative Analysis of Cytokines from Single Macrophages
Source: ACS Nano Med. 2026 Mar 3;1(5):1227–38. doi: 10.1021/acsnanomed.5c00149 (PMC13262127; doi:10.1021/acsnanomed.5c00149)
Supplement: Supplementary file 1 [file nm5c00149_si_001.pdf]

Supporting Information

Nested Nanowell Arrays for High-Throughput Quantitative Analysis of Cytokines from Single Macrophages

*Claudius L. Dietsche, Lucien R. Stöcklin, Robert Strutt, and Petra S. Dittrich**

Department of Biosystems and Engineering, ETH Zurich, Basel, SWITZERLAND

Author information:

Claudius L. Dietsche,	claudius.dietsche@bsse.ethz.ch	*0000-0003-3732-6971*
Lucien L. Stöcklin,	lucien.stoecklin@bsse.ethz.ch	*0009-0000-5428-0210*
Robert Strutt,	robert.strutt@bsse.ethz.ch	*0000-0003-0979-2000*
Petra S. Dittrich*	petra.dittrich@bsse.ethz.ch	*0000-0001-5359-8403*

***Corresponding author:**

Petra S. Dittrich
ETH Zürich
Schanzenstrasse 44
CH-4056 Basel/Switzerland

e-mail: petra.dittrich@bsse.ethz.ch

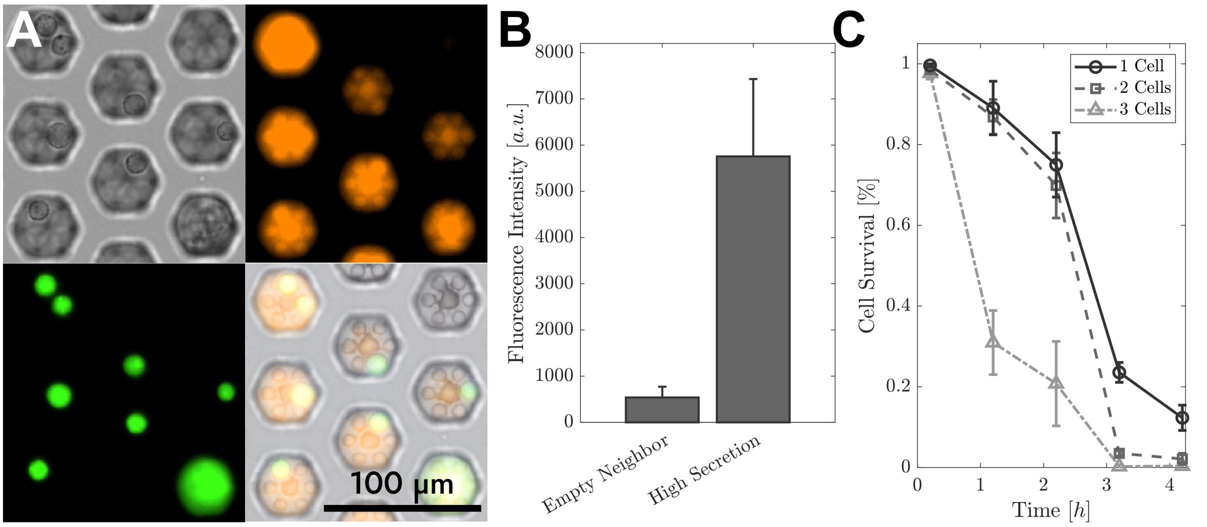

Figure S1. Additional characterization. (A) Example image of Calcein AM stained cells in media containing alamarBlue™. An empty well can be seen in the top right corner. In the lower left corner, the Calcein AM leaked out of the cell indicating a dead cell. (B) Selected are the wells containing the 2% highest secreting cells for IL-8 and their empty neighbor wells. The readout in the empty wells is below the detection limit showing no cross-contamination from one well to the other. (C) Cell survival over a period of 4 hours depending on the number of cells in each well ($n_{1Cells} \geq 2191$, $n_{2Cells} \geq 316$, $n_{3Cells} \geq 26$).

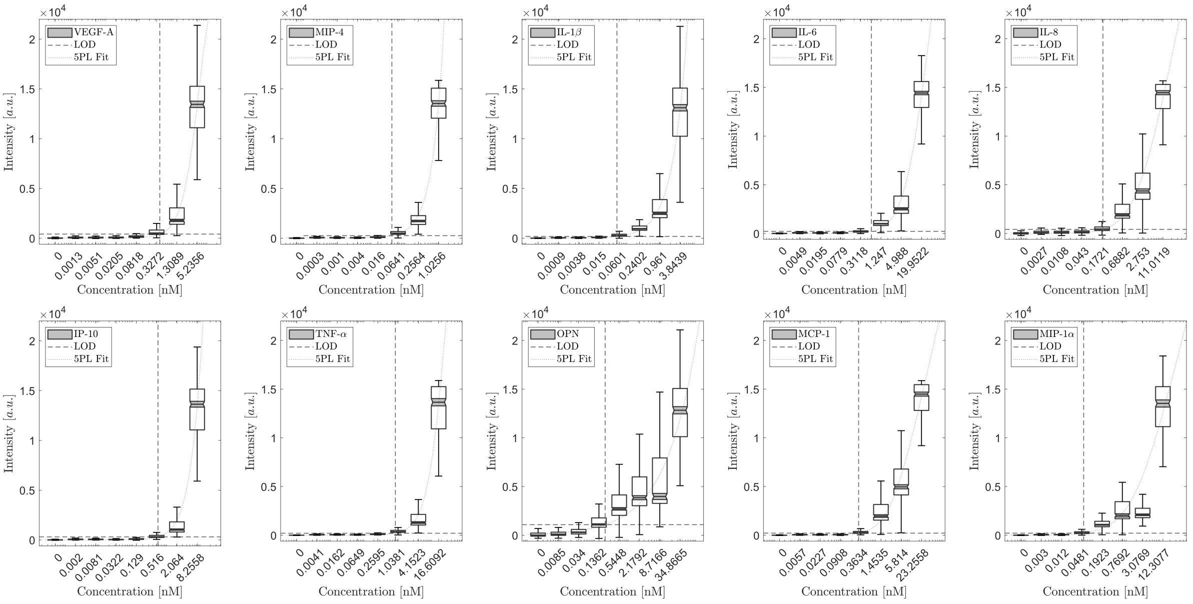

Protein	VEGF-A	MIP-4	IL-1β	IL-6	IL-8	IP-10	TNF-α	OPN	MCP-1	MIP-1α
LOD Concentration [ng/mL]	14.66	0.38	1.22	8.639	1.538	4.85	21.32	5.43	2.36	0.45
LOD Concentration [nM]	0.406	0.042	0.051	0.649	0.182	0.566	0.864	0.208	0.316	0.055
LOD [# Molecules]	14’626	1’860	2’693	15’754	6978	21’475	46’708	6’142	10’457	2’212

Figure S2. Calibration curves for all proteins analysed. The limit of detection (LOD) was calculated by adding 3 times the standard deviation to the median value of the control (0 nM). The 5PL Fit was fitted with MATLAB. For the fit, the concentrations around the LOD were more heavily weighted to increase the precision of the fit around the LOD. A volume of 63.29 picolitres was used to calculate the number of molecules in each well at the LOD.

$$C_k(n) = \binom{n+k-1}{k} = \frac{(n+k-1)!}{k!\,(n-1)!}$$

Figure S3. Equation to determine the possible combinations in our wells-in-well array. Here, n represents the number of different bead types (10) investigated and k represents the number of wells for magnetic beads (7). The equation accounts for the possibility of having more than one bead of the same type in one well.

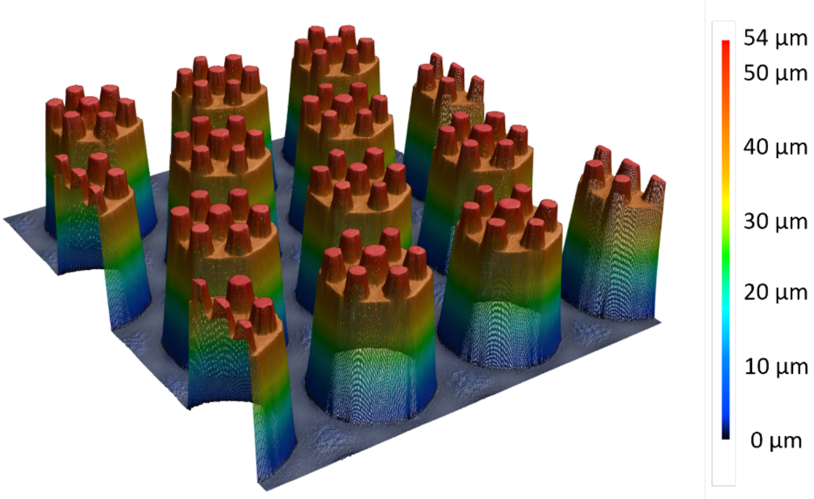

Figure S4. Microscopic image of the master mould wafer with its SU8 structures. The image was taken with a 3D laser scanning microscope (Keyence VK-X3000). The height of the hexagonal wells is ~44 µm and the height of the magnetic bead traps is ~9 µm.

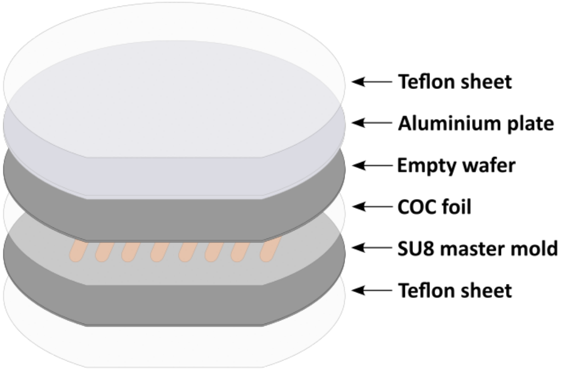

Figure S5. Fabrication process of the COC device. Two Teflon sheets prevent sticking of the master mold and the aluminium plate to the CNI tool. The COC foil is heated between the master mould and an empty and silanized wafer. The aluminium plate is needed to prevent breaking of the silicon wafers and apply uniform pressure to the SU8 master mold.

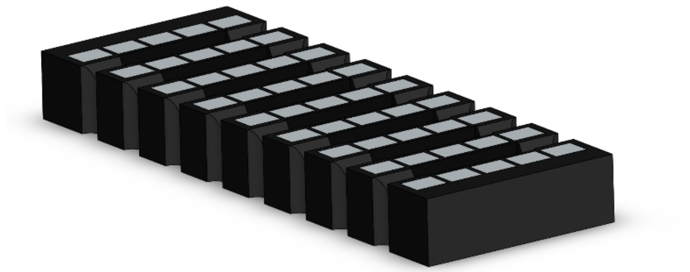

Figure S6. CAD model (Solidworks 2021) of the magnet holder. Five magnets are placed on each side of the eight conditions (45 in total). Due to the placement of the magnets, the microwell array is located in a strong magnetic field pulling the magnetic beads downwards without blocking the view for brightfield imaging.

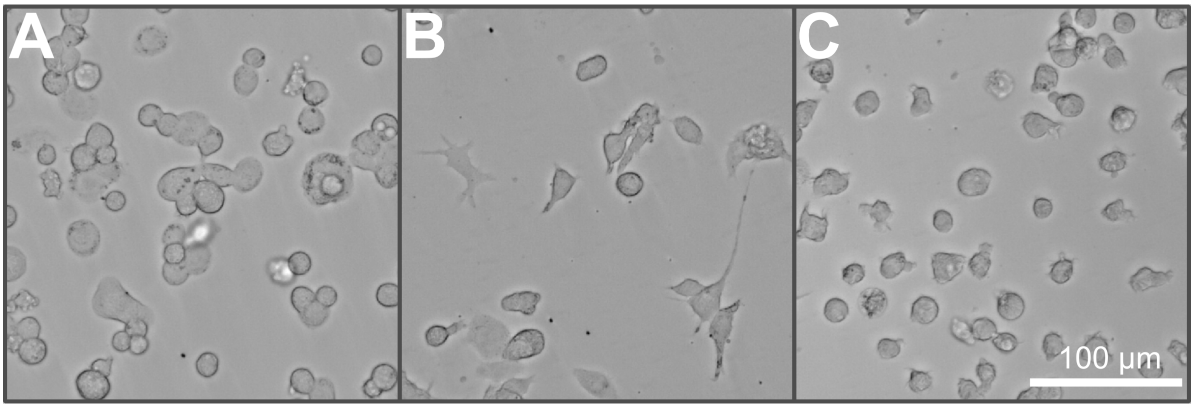

Figure S7. Microscopic images of differently stimulated macrophages. (A) THP-1 cells were incubated in 100 ng/mL PMA for 24h (MΦ). (B) After incubation with PMA for 24h, LPS (1 µg/mL) and IFN-γ (50 ng/mL) were added for 12 h (MΦ+LPS/IFN-γ). (C) IL-4 (50 ng/mL) and IL-13 (50 ng/mL) were added after 24 h for 12 h (MΦ+IL-4/IL-13). Scale bar: 100 µm

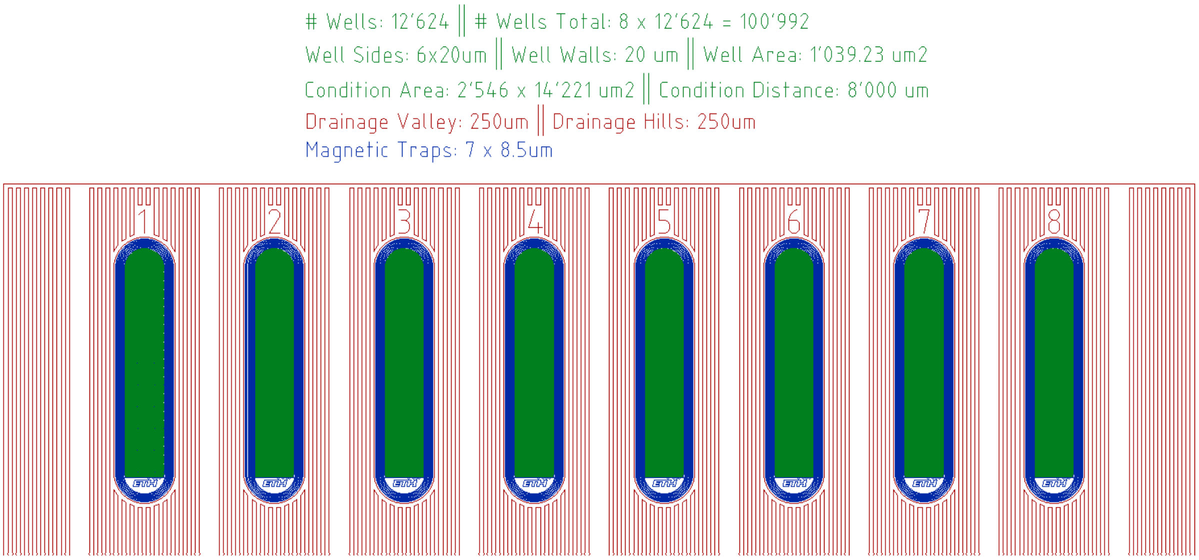

Figure S8. The soft lithography mask design. The drainage system is shown in red, designed to prevent cross-contamination between different conditions by directing excess liquid to the sides of the platform. Blue represents the channels surrounding each condition, which help contain the liquid during pipetting. Green highlights the areas of the wells-in-well.

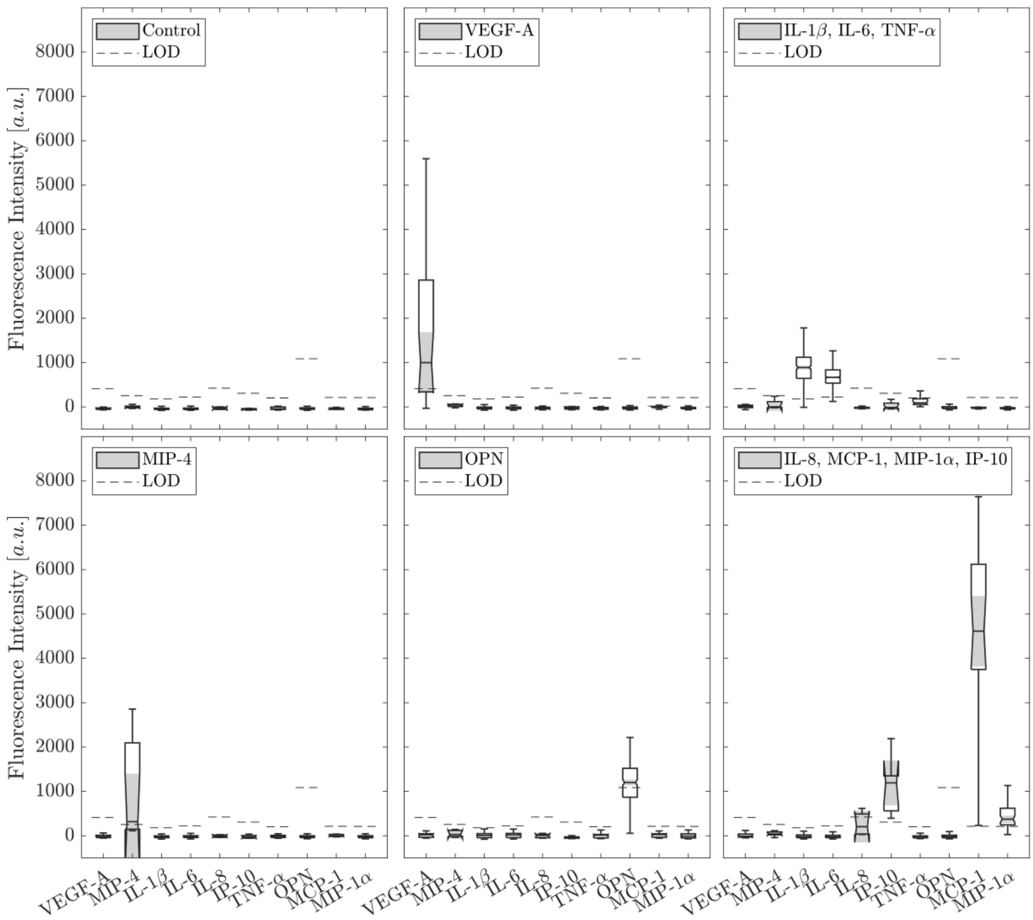

Figure S9. Crosstalk of the target proteins. The different protein suspensions provided by the ProcartaPlex^{TM} assay kit were tested for cross-reactivity of the different proteins. The experiments were conducted on a 96 well plate according to the assay protocol.

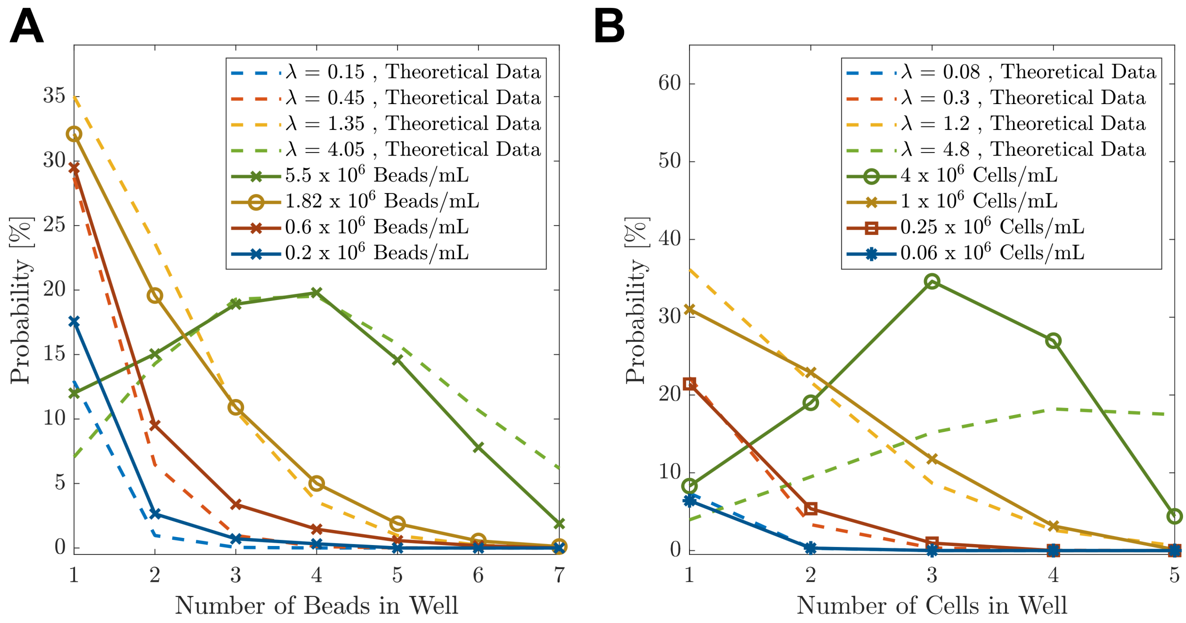

Figure S10. Number of beads (A) and cells (B) per well compared to theoretically calculated values based on the Poisson distribution. For the bead distribution, the theoretical predictions and empirically measured values are in good agreement. For the cell distribution, good agreement is observed at lower concentrations; however, due to size limitations, the theoretical and empirical measurements begin to deviate at higher concentrations.

Table S1. Detailed information on the proteins and chemicals used in the cell experiments.

Product	Supplier	Article Number	Source/CAS-Nr.
Recombinant Human IFN-γ	Sigma Aldrich	SRP3058	*E.Coli*
Recombinant Human IL-4	PeproTech	200-04	*E.Coli*
Recombinant Human IL-13	Thermo Fisher Scientific	A42526	CHO cells
Phorbol 12-myristate 13-acetate (PMA)	Thermo Fisher Scientific	J63916.MCR	16561-29-8
Lipopolysaccharide (LPS)	Thermo Fisher Scientific	00-4976-03	*Escherichia coli* 026:B6
Paclitaxel	Sigma Aldrich	Y0000698	33069-62-4
Anhydrous Docetaxel	Sigma Aldrich	Y0001466	114977-28-5

Table S2. Detailed information on the ProcartaPlex^{TM} immunoassays used during all experiments. All products were ordered from Thermo Fisher Scientific.

Product	Article Number	LOT	Comment
IL-1β	EPX01A-10224-901	312727-005, 312727-005	Included: Beads, Detection Antibody, Protein Standard
IL-8	EPX01A-10204-901	341865-007, 341865-011	Included: Beads, Detection Antibody, Protein Standard
MIP-1α	EPX01B-12029-901	359163-007, 374555-004	Included: Beads, Detection Antibody, Protein Standard
TNF-α	EPX01A-10223-901	350092-002, 350092-004	Included: Beads, Detection Antibody, Protein Standard
VEGF-A	EPX01A-10277-901	355048-001	Included: Beads, Detection Antibody, Protein Standard
OPN	EPX01A-12066-901	377648-002	Included: Beads, Detection Antibody, Protein Standard
MCP-1	EPX01B-10281-901	377056-001	Included: Beads, Detection Antibody, Protein Standard
MIP-4	EPX010-12383-901	309184-005	Included: Beads, Detection Antibody, Protein Standard
IP-10	EPX01A-10284-901	364839-001	Included: Beads, Detection Antibody, Protein Standard
IL-6	EPX01A-10213-901	360354-001	Included: Beads, Detection Antibody, Protein Standard
Basic Kit	EPX010-10420-901	313843-000	Included: Reading Buffer, Washing Buffer, Streptavidin-PE, Detection Ab Diluent
